# A comparison of alternative selection methods for reporting spirometric parameters in healthy adults

**DOI:** 10.1038/s41598-021-94120-9

**Published:** 2021-07-22

**Authors:** Jennifer H. Therkorn, Daniella R. Toto, Michael J. Falvo

**Affiliations:** 1 Airborne Hazards and Burn Pits Center of Excellence, War Related Illness and Injury Study Center, VA New Jersey Health Care System, East Orange, NJ USA; 2 Rowan University School of Osteopathic Medicine, Stratford, NJ USA; 3 Departments of Pharmacology, Physiology and Neuroscience and Physical Medicine and Rehabilitation, Rutgers New Jersey Medical School, Newark, NJ USA

**Keywords:** Respiration, Translational research

## Abstract

Alternative methods have been proposed to report spirometry indices from test sessions (forced expiratory volume 1 s, FEV_1_; forced vital capacity, FVC). However, most use the American and European Societies’ standard (ATS/ERS) which stops sessions once a repeatability threshold is met which may not accurately represent intra-session variability. Our goal was to repeat trials beyond the repeatability threshold and evaluate alternative reporting methods. 130 adults performed spirometry across two visits. Spirometry indices were reported using the ATS/ERS standard and four alternatives. 78 participants (60%) had valid data for all methods and visits. Intra-session coefficients of variation were low (FEV_1_: 3.1–3.7%; FVC: 2.3–2.8%). Our four alternative methods yielded FEV_1_ and FVC values ≤ 0.08 L different from ATS/ERS standard, which is not clinically meaningful. Intraclass correlation coefficients were ≥ 0.97 indicating consistency across repeated measures. The smallest real differences ranged from FEV_1_: 0.20–0.27 L and FVC: 0.18–0.24 L indicating consistency and low measurement error. Overall, all methods for reporting FEV_1_ and FVC demonstrated similar measurement error, precision, and stability within- and between-visits. These results suggest that once ATS/ERS repeatability is achieved, which approach is used for reporting spirometric variables may be of low clinical significance in a healthy population.

## Introduction

Results of spirometry testing are of substantial consequence in clinical, occupational, research and policy settings. Spirometry is often the initial test performed for individuals with respiratory symptoms and is used to characterize disease severity, monitor and optimize therapy, as well as guide clinical decision making during preoperative evaluation^1^. In workplaces with known or suspected respiratory hazards, spirometry is a central feature of medical surveillance programs to prevent respiratory disease and protect workers^2^. For these reasons, spirometric variables—e.g., forced expiratory volume in 1 s (FEV_1_)—are frequently used as the primary endpoint in many clinical research trials. Additionally, controlled human exposure research studies using varying levels of air pollutants and evaluating the acute changes in spirometry before and after exposure are considered essential to understanding the dose–response relationship of associated health effects^3^.

At present, spirometry is most widely performed and reported in accordance with the American Thoracic and European Respiratory Societies (ATS/ERS) recommendations^4^. The process for reporting spirometric variables for a given test session follows a series of checkpoints: (1) confirming technical acceptability of each trial or maneuver performed in a test session^4^, (2) achieving the repeatability criteria with respect to all trials from a test session, and (3) reporting a single value for each spirometric variable to represent that test session^4^. The ATS/ERS method calls for a minimum of three and maximum of eight maneuvers within a test session. Test termination occurs when repeatability criteria are met—i.e., the two largest forced vital capacity (FVC) and FEV_1_ values from acceptable maneuvers are ≤ 0.150 L—resulting in a range of 3–8 maneuvers that may comprise a test session. FVC and FEV_1_ values reported are the largest from a single trial and all other values from maneuvers are discarded. Surprisingly, the current ATS/ERS method remains remarkably similar to the initial description of spirometry over 175 years ago—i.e., “Let the observation for measuring the forced expiration be made three times, and the greatest of them be noted as the vital capacity” (pg. 241–242^5^).

In selecting the largest FVC and FEV_1_ values to report, the ATS/ERS method has been justified by its ability to account for the influence of learning or fatigue effects^5^. However, terminating testing following achievement of repeatability criteria precludes the ability to evaluate a learning effect. In addition, the assumption of a deterioration in performance due to fatigue is contradictory to reported observations^6^. Belzer and Lewis^7^ recently questioned these assumptions and highlighted their implications on measurement error. The authors illustrated the apparent paradox of the ATS/ERS method which recommends performing multiple maneuvers, presumably to account for within-subject or intra-session variability, while simultaneously terminating test sessions once repeatability criteria are achieved. The latter precludes the quantification of intra-session variability, or critical information regarding test and biological variation.

Performance of multiple maneuvers to report only the single largest FVC and FEV_1_ values as opposed to the mean has previously been debated within the ATS^8^. The optimal method for selecting how to report a test session’s spirometric variables likely depends on the specific question being asked as well as the setting (i.e., clinical versus research^9,10^); however, a selection method that seeks to minimize both biologic and measurement variability would seem most appropriate^11^. It is important to minimize variability in FEV_1_ and FVC measurements both within and between test sessions therefore allowing the reported spirometric variables from a single test session to be most representative of that test session; this has wider implications. For example, reducing noise can provide future studies greater power to detect treatment effects and enable better identification of important clinical outcomes across visits^12^. Many studies have observed reduced variability when using the mean in comparison to maximum values^9,10,13–17^, though this finding is not universal^18–21^. Of note, these now decades old studies focused almost exclusively on between- or inter-session variability, included ≤ 5 total maneuvers in a test session, and often restricted analysis to FEV_1_. The latter is of particular importance as the largest FEV_1_ from a set of maneuvers for many individuals is submaximal due to thoracic gas compression^18^, hence why other investigators recommend reporting values from the maneuver with the greatest peak flow^22,23^. Despite these unresolved questions, the ATS/ERS method remains widely used across clinical and research settings.

With the advent of newer spirometry technology and recent changes to acceptability criteria (i.e., end of forced expiration criteria) and calls for greater standardization of measurement^4^, there is a need to re-evaluate our current variable selection and reporting approaches with greater attention to the multiple maneuvers available for analysis when test sessions are not terminated once repeatability criteria are achieved^24^. The overarching question is whether or not alternative methods for selecting and reporting spirometric indices can provide more representative results of the test session as compared to the ATS/ERS standard. This question can only be answered by collecting data beyond the point where repeatability is first achieved to understand intra-session variability after crossing the repeatability threshold. Therefore, the purpose of this study was to acquire spirometry data under rigorously controlled conditions to determine which selection method of spirometric maneuvers would yield the most precise and repeatable measures in a healthy adult population. As each participant performed at least eight repeated FVC maneuvers, on two separate occasions, the present study afforded determination of intra- and inter-session variability of key spirometric variables. We hypothesized that selection methods to report spirometric indices that were not restricted to only including trials up to when repeatability was first established would confer less variability between test session visits. The rationale behind this hypothesis is that achieving repeatability results in reduced intra-session variability that is best reflected by trials performed beyond the repeatability threshold.

## Methods

### Participants

We recruited 137 adults between the ages of 18–40 years to participate in this study. Recruitment efforts included e-mail distribution to the university community, posting flyers across multiple campuses and community centers, internet advertisement (i.e., ResearchMatch.org, laboratory website) and word of mouth. Participants had no recent history of respiratory illness or infection (≤ 3 weeks) and had refrained from recent vigorous exercise (≤ 24 h). Exclusion criteria included: (1) any contraindication to spirometry^4^, (2) use of traditional tobacco products within the past 12 months (former smokers with lifetime pack-year history ≤ 5 years may be included), (3) e-cigarette/vaping or smokeless tobacco use ≥ once per month, (4) history of cancer or major organ disease, (5) childhood history of asthma, (6) current use of medications known to affect lung function (e.g., bronchodilator medications, methotrexate, amiodarone, statins), (7) pregnant, or (8) morbidly obese (≥ 40 kg/m^2^). All participants provided their written informed consent and study procedures were approved by the Rutgers University Institutional Review Board. All research was performed in accordance with relevant guidelines and regulations, including all ethical principles for research involving human subjects.

### Experimental design

The study required participants to attend two laboratory sessions separated by 7 days (± 3 days) and performed at the same time of day (± 2 h). At the first visit, participant demographic and anthropometric characteristics were obtained. Height (stocking feet) and weight were measured using a portable stadiometer (Seca, Germany) and digital scale, respectively. Waist and hip circumference were measured using a Gulick tape measure to calculate the waist to hip ratio. Waist and hip circumferences were measured at the height of the iliac crest and at the widest circumference of the buttocks, respectively^25^. Prior to testing, participants completed a series of surveys using REDCap electronic data capture tools hosted at Rutgers University^26,27^. Internally developed and standardized surveys were used to assess prior experience with breathing tests, physical activity history, sleep quality, and mood disturbance. Physical activity was quantified by computing metabolic equivalent minutes (MET minutes) per week from the International Physical Activity Questionnaire^28^. Sleep quality was determined from the Pittsburgh Sleep Quality Index and computation of a global score^29^. Total mood disturbance was calculated from the 30-item Short Form of the Profile of Mood States^30^. Prior to testing, participants were asked, “How well do you expect to perform on today’s breathing maneuvers?” and responded on a 7-item Likert-based scale (1 = extremely poor, 7 = excellent). Personal smoking history (traditional tobacco products, e-cigarettes/vaping, smokeless tobacco) and second-hand smoke exposure at home and at work were also obtained by questionnaire. These variables were collected both to characterize our sample as well as to identify potential predictors of intra- or inter-session variability. At the end of each visit, participants were asked to rate their ‘comfort’ and ‘ease’ with spirometry maneuvers using a visual analog scale (0–100) with 100 indicating ‘very comfortable’ and ‘very easy’, respectively.

### Spirometry protocol

A single trained technician, who had completed a NIOSH-approved spirometry course prior to enrollment, performed all data acquisition. Prior to testing, participants were read a standardized script of instructions and provided a physical demonstration of proper spirometry technique. All testing was performed using a flow-type spirometer (Easy-on PC spirometer; nDD Technologies, Zurich, Switzerland) that was secured to a flexible arm device (Gooseneck Holder LS06; Lamicall). The flexible arm enabled the spirometer to be individually positioned by the technician to achieve proper positioning (e.g., chin slightly elevated). Testing was performed in a seated position with hands relaxed on lap while wearing a nose clip. A calibration check was performed prior to each test at three flow rates using a 3-L calibrated syringe to ensure accuracy. Biological control testing was also performed quarterly.

For each maneuver, the following sequence was followed: (1) tidal breathing, (2) maximal inspiration, (3) maximal exhalation, and (4) maximal inspiration. Participants initiated the first maximal inspiration on their own volition but cueing of the final inspiration was instructed by the technician corresponding to expiratory plateau. Eight maneuvers—or trials—were consecutively obtained with an inter-trial rest interval of approximately 60 s. In certain circumstances, the technician may prematurely terminate a maneuver in the event of zero-flow error, participant miscuing and/or software error. If a trial was prematurely terminated by either the technician or participant, additional trials were performed to reach a total of 8 trials with a maximum of 16 trials. Errors detected in real-time (e.g., early termination, submaximal inhalation) were identified by the technician and coaching corrections were made in accordance with NIOSH recommendations^31^.

### Data quality and reduction

Each trial was individually inspected by a single investigator, a NIOSH-approved spirometry course director, to evaluate acceptability. Using established criteria for acceptability^4^ each trial was inspected to ensure: (1) forced inspiratory vital capacity [FIVC] – FVC ≤ 100 mL or 5% of FVC, (2) back extrapolated volume ≤ 5% of FVC or 100 mL, (3) expiratory plateau, and (4) absence of cough, glottis closure, leak and/or zero-flow error. Failure to meet any of these four criteria resulted in the trial being marked as unacceptable and excluded from subsequent analysis. The type of error(s) violating acceptability criteria was noted by the investigator in the software program and indicated in a spreadsheet.

### Selection methods

We compared five methods for the selection of how to report FEV_1_ and FVC from test sessions. These included the following: (1) ATS/ERS^4^, (2) modified ATS/ERS method, (3) mean of all maneuvers^6^, (4) mean of three largest values^15^, and (5) peak effort^23^ (Table 1). Other spirometric parameters—i.e., peak expiratory flow and mid-expiratory flow—are summarized in the Supplemental Materials.

**Table 1:** Selection methods for forced vital capacity (FVC) and forced expiratory volume in one second (FEV_1_) indices for a spirometry session.

Selection method	Repeatability criteria	Total number of maneuvers considered to establish repeatability (intra-session comparison)	Selection of single FVC and FEV_1_ from repeatable maneuvers (inter-session comparison)	Selection of PEF and FEF_25–75_ from repeatable maneuvers (inter-session comparison)
1. ATS/ERS	Largest and second largest FVC and FEV_1_ ≤ 0.15 L	Rolling inclusion of trials until repeatability achieved (at least 3)	Largest	Best sum of FVC and FEV_1_
2. Modified ATS/ERS	Largest and second largest FVC and FEV_1_ ≤ 0.15 L	Rolling inclusion of trials until repeatability achieved (at least 3)	Mean	Mean
3. Mean of all	Largest and second largest FVC and FEV_1_ ≤ 0.15 L	All	Mean	Mean
4. Mean of three largest	Largest and second largest FVC and FEV_1_ ≤ 0.15 L	All	Mean of three largest	Mean for three largest sums of FVC and FEV_1_
5. Peak effort	Largest and second largest PEF ≤ 10%	All	Trial with largest PEF	Trial with largest PEF

ATS/ERS refers to the current selection method recommended by the American Thoracic and European Respiratory Societies. The parameters PEF (peak expiratory flow) and FEF_25–75_ (forced mid-expiratory flow) are reported in the Supplemental Materials.

#### Selection of trials for intra-session comparison

The methods for trial selection began by establishing the trials that would be considered for a given subject and visit to meet repeatability criteria and then it was determined whether that subject met repeatability. For all selection methods, any subject not having at least two acceptable maneuvers were removed from further consideration. For the ATS/ERS and modified ATS/ERS methods, for each subject and visit, trials were assessed for repeatability after at least three trials were performed up to and including the maneuver at which repeatability was first established; repeatability was defined as a subject achieving a difference across maneuvers’ maximum and second maximum for FEV_1_ and FVC less than or equal to 0.15 L. In contrast, for the selection methods mean of all maneuvers and mean of three largest values, the repeatability definition was the same, but the trials considered for establishing repeatability included all acceptable trials for a given subject and visit. For the peak effort selection method, the trials considered for establishing repeatability also included all acceptable trials for a given subject and visit, but with the repeatability definition being that the percent difference between the maximum and second maximum for PEF had to be less than or equal to 10%. (Sample R code for trial selection is provided in the Supplemental Materials).

#### Reporting of indices for inter-session comparison

Following the selection of maneuvers to include for intra-session comparison, the goal was to select a single value for FEV_1_ and FVC from these maneuvers for each subject and visit for inter-session comparison. For the ATS/ERS method, FEV_1_ and FVC were chosen as the maximum values across a subject’s included maneuvers. For the modified ATS/ERS method and the mean of all method, FEV_1_ and FVC were taken as the mean across included maneuvers. The mean of three largest method used an approach that combined both of these selection methods where the three largest FEV_1_ and FVC values were selected from each subject’s included maneuvers and then the mean taken for these values. Finally, for the peak effort selection method, FEV_1_ and FVC were taken from the maneuver where PEF was at its maximum.

### Statistical analysis

To evaluate intra-session variability for FEV_1_ and FVC, we compared all maneuvers meeting repeatability criteria for each selection method during each visit. The study design incorporated repeat measures where each subject was supposed to attend two testing sessions; however, not all subjects were able to complete both testing sessions nor able to achieve repeatable data across all selection methods. For these reasons, it was determined that a linear mixed effect model was most appropriate for the present dataset^32^. A mixed effect model^33^ was fit to the data with FEV_1_ or FVC as the response variable. Subject number was included in the model as a random effect. The purpose for assigning this variable as a random effect in the model was to account for the non-independence of multiple measurements from each individual subject^32^. The measurement error for the mixed effect model fit was taken as the root mean square error (i.e., square root of the variance of the residuals). This is interpreted as the common within subject standard deviation (SD), or measurement error^34^. SD was determined for each model fit after delimiting data between the two visits and each selection method. Measurement precision was then assessed by the coefficient of variation (CV%; CV% = SD/mean * 100)^34^. Intra-session repeatability was calculated by multiplying SD and 2.77 (√2 × 1.96)^35^, referred to as the smallest real difference (SRD). SRD% was calculated as the ratio of SRD to the overall mean of the measurement value across both visits multiplied by 100.

To evaluate inter-session variability, a single value of FEV_1_ and FVC was selected for each subject and visit. A mixed effects model was fit to the data with FEV_1_ or FVC as the response variable after delimiting data for each selection criteria. Subject number was included in the model as a random effect and visit number was included as a fixed effect. Similarly, as for the intra session variability calculations, the SD for the mixed effect model fit has been taken as the root mean square error and used to calculate SRD, SRD% and CV%. Intraclass correlation coefficients (ICC) were calculated via a two-way mixed effects model for absolute agreement from single measurements, with 95% CIs around absolute agreement^36^. Bland Altman plots are provided in Supplemental Materials Figure S2 and Table S7 to report biases and limits of agreement with 95% confidence intervals (CIs)^37^. Participant and behavior characteristics were compared between visits using Wilcoxon signed-rank tests and between those with and without valid data using Mann Whitney U tests. Analyses were performed and figures created using R statistical computing software (R version 4.0.2, June 2020;^38,39^).

Sample size was determined a priori using the methodology for constructing precise confidence intervals around the ICC measurement^36,40^. In this case, for the primary outcome of ICC using spirometry, eight repeated measures (i.e., spirometry maneuvers), with a planned ICC estimate of 0.90 (based on prior literature^13^), a precision of ± 3.0% for confidence interval width, using a two-sided significance level (α) of 0.05, and an assumed maximum of 15% drop-out, 97 participants were estimated to properly power the study.

## Results

### Participants

Of the 137 participants enrolled, four individuals were excluded post-consent after meeting one of our exclusion criteria (morbid obesity, n = 2; pregnant, n = 1; e-cigarette/vaping use ≥ monthly, n = 1) and three participants were unable to perform acceptable maneuvers on either visit. Five participants completed Visit 1 only (respiratory infection after Visit 1, n = 1; vigorous exercise ≤ 24 h prior to Visit 2 = 1; administrative hold due to SARS-CoV-2 pandemic, n = 3). Participant characteristics are reported in Table 2 for those with acceptable maneuvers on either visit (n = 130).

**Table 2:** Participant characteristics.

Participant characteristics	N = 130
Age	25.6 (18–39)
**Anthropometrics**	
Body mass index (kg/m^2^)	24.9 (17.6–39.9)
Waist/hip ratio	0.78 (0.66–1.05)
**Sex**	
Male	37, 28.5%
Female	93, 71.5%
**Race**	
Asian	40, 30.8%
Black	20, 15.4%
White	57, 43.8%
Multi-racial	7, 5.4%
Unknown	6, 4.6%
**Ethnicity**^**a**^	
Hispanic or Latino	18, 14.1%
Non-Hispanic or Non-Latino	110, 85.9%
**Education level**	
Some college	12, 9.2%
Undergraduate degree	42, 32.3%
Graduate/professional degree	76, 58.5%
**Smoking history**	
≥ 100 cigarettes lifetime (n, %)	1, 0.8%
Pack-year history (mean, range)	0.03 (0.0–0.05)
**E-cigarette, vaping**	
Ever use (n, %)	28, 21.5%
Occasional use (n, %)	8, 6.2%
**Smokeless tobacco**	
Ever use (n, %)	1, 1.5%
Occasional use (n, %)	0, 0.0%
**Secondhand smoke exposure**	
At work	5, 45.5%
At home	4, 3.1%

Values are presented as mean (range), or frequency and percentage.

^a^Missing data (n = 2).

Approximately 26.9% of our sample (n = 35) had previously performed at least one breathing test prior to this study, including 12 participants (34.3%) who had performed breathing tests up to four times. The majority (80.0%) had last performed a breathing test ≥ 6 months prior to their study visit. Approximately 39.2% had regularly engaged (≥ 6 months) in respiratory focused breathing exercises (power/weightlifting, 35.4%; yoga breathing exercise, 24.6%; swimming, 16.9%; other breathing exercise, 6.9%; inspiratory muscle training, 0.8%), and approximately 43.0% met the minimum (≥ 600 MET minutes) recommended physical activity levels per week (1393.7 ± 1735.6 MET min wk^−1^). Sleep quality (Visit 1: 6.79 ± 2.19, Visit 2: 6.63 ± 2.12; Wilcoxon signed rank; Z = − 1.05, *p* = 0.295), total mood disturbance (Visit 1: 21.55 ± 8.36, Visit 2: 21.13 ± 8.95; Z = − 0.36, *p* = 0.721) and pre-test performance expectation (Visit 1: 0.96 ± 1.03, Visit 2: 0.97 ± 1.05; Z = − 0.15, *p* = 0.879) were all similar between visits. When asked how comfortable and easy the spirometry maneuvers were to complete following each visit, participants rated a similar level of comfort on both visits (Z = − 1.21, *p* = 0.225) but endorsed greater ease in performing spirometry maneuvers on Visit 2 relative to Visit 1 (Z = − 2.46, *p* = 0.014). Prior to testing, approximately 33% (Visit 1) and 32% (Visit 2) expected to perform ‘very good’ or ‘excellent’ on breathing maneuvers. Smoking history and exposure are also reported in Table 2.

### Session and trial characteristics

**Figure 1:**
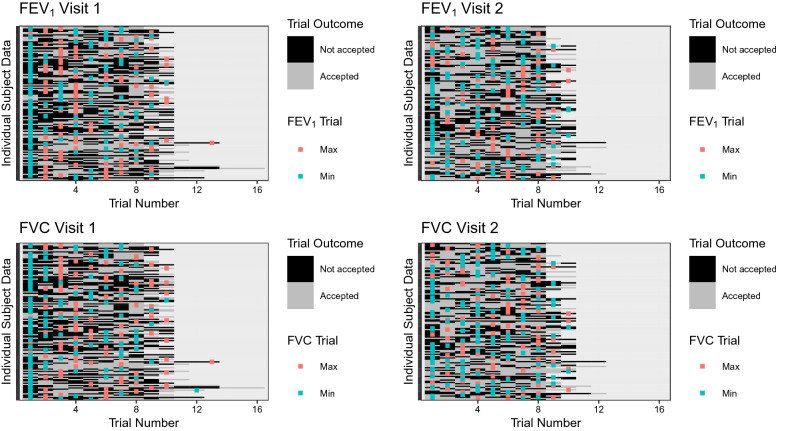
Heat map diagram illustrating the minimum and maximum forced expiratory volume in one second (FEV_1_) and forced vital capacity (FVC) vs. trial outcome (accept/not accept) for each visit and subject. Data are displayed across the vertical axis for each individual subject. The outcomes for every subject’s attempted trial (accepted vs. not accepted) are represented by the black and grey boxes with trial number indicated on the horizontal axis. The absolute maximum and minimum value for each subject’s FEV_1_ and FVC measurement are indicated with a red and blue marker, respectively.

Table 3 describes participant performance across visits including the number of acceptable and valid trials as well as the frequency of common errors. Median inter-visit duration was 6.1 days (lower quartile, upper quartile [IQR]: 6.0, 7.0). Overall, 2370 maneuvers were performed and 51.5% were accepted. Failure to achieve a plateau was the most common error type (22.7%). Within each visit, ≥ 80% of our subjects were able to achieve valid data defined as meeting acceptability and repeatability criteria. However, 78 of 130 (60.0%) had valid data for all five selection methods on both days. In Fig. 1, we plotted the occurrence of minimum and maximum values of FEV_1_ and FVC per participant and session. This data represents all maneuvers attempted.

**Table 3:** Visit and test characteristics.

Characteristic	Visit 1	Visit 2	Overall (both visits)
Total maneuvers, n	1230	1140	2370
Acceptable, n (%)	593 (48.2)	628 (55.1)	1221 (51.5)
Number of subjects with acceptable maneuvers, n (%)	124 (93)	123 (96)	117 (88)
**Number of subjects with valid maneuvers, n (%)**			
ATS/ERS (standard and mean)	99 (80)	110 (89)	90 (69)
Mean of all and three largest	99 (80)	110 (89)	90 (69)
Peak effort	100 (81)	109 (89)	87 (67)
Across all selection methods	107 (86)	115 (93)	78 (60)
**Total number of maneuvers per subject**			
Mean ± SD (range)	9 ± 1 (6–16)	9 ± 1 (8–13)	9 ± 1 (6–16)
Median (IQR)	9 (8, 10)	9 (8, 10)	9 (8, 10)
**Total number of accepted maneuvers per subject**			
Mean ± SD (range)	5 ± 2 (1–10)	5 ± 2 (1–10)	5 ± 2 (1–10)
Median (IQR)	5 (4, 6)	5 (4, 7)	5 (4, 7)
**Percent (%) of accepted maneuvers per subject**			
Median (IQR)	60.0 (44.4, 75.0)	66.7 (55.6, 80.0)	62.5 (50.0, 77.8)
**Unacceptable maneuver error type, n (%)**			
No plateau	239 (19.4)	251 (22.0)	538 (22.7)
Early termination	80 (6.5)	27 (2.4)	107 (4.5)
Breath hold/glottis closure	57 (4.6)	40 (3.5)	97 (4.1)
Leak	77 (6.3)	81 (7.1)	156 (6.6)
Submaximal inhalation	41 (3.3)	35 (3.1)	76 (3.2)
Cough	17 (1.4)	15 (1.3)	33 (1.4)
Variable effort	26 (2.1)	11 (1.0)	38 (1.6)
Other	47 (3.8)	24 (2.1)	71 (3.0)
Multiple	17 (1.4)	10 (0.9)	26 (1.1)

ATS/ERS refers to the current selection method recommended by the American Thoracic and European Respiratory Societies. Data are presented as mean ± 1 standard deviation (SD), median (interquartile range, IQR) or frequency and percentage.

Participant characteristics were similar between those with valid (n = 78) and invalid data (n = 57) for age, height, sex, race, ethnicity, smoking history, physical activity, sleep quality, total mood disturbance and performance expectation on either visit (*p* > 0.05; data not shown). Participants with complete data had greater body mass (Valid vs. invalid; 73.9 ± 17.0 vs. 68.0 ± 19.8 kg, *p* = 0.021), body mass index (25.9 ± 4.8 vs. 23.9 ± 5.9 kg/m^2^, *p* = 0.002) and waist-to-hip ratio (0.79 ± 0.07 vs. 0.77 ± 0.07, *p* = 0.021) than those with incomplete data. Intra- and inter-visit variability analyses presented below were delimited to a primary dataset consisting of those with valid data across both visits and all selection methods (n = 78). However, a complete report of all intra- and inter-session analyses for datasets with subjects not having valid data across sessions and selection methods can be found in the Supplementary Materials. These secondary analyses yielded similar results suggesting the decision to proceed with the chosen primary analysis dataset did not impact our results or interpretations.

### Intra-session variability

Descriptive statistics are provided in Table 4 reflecting the corresponding trials for each of the five selection methods. This phase of trial selection was based on selecting all trials meeting repeatability criteria for each subject and visit; therefore, multiple criteria share the same selected repeatable trials as described in the repeatability definitions in Table 1. To investigate which selection method produces the least short-term intra-visit variability in outcome measures (FEV_1_ and FVC), a mixed effect model was fit to the data with FEV_1_ or FVC as the response variables after delimiting data between the two visits and each selection criteria. The ranges observed for measurement error (SD), SRD and CV% for FEV_1_ and FVC were similar across selection methods and visits (Table 4) with a tendency toward reduced variability in Visit 2.

**Table 4:** Intra-session descriptive statistics and variability by selection method for forced vital capacity (FVC) and forced expiratory volume in one second (FEV_1_) (n = 78 subjects).

Selection method	Variable	Visit 1							Visit 2						
		n	Median (IQR) (L)	Mean ± SD (L)	SD (L)	SRD (L)	SRD%	CV%	n	Median (IQR) (L)	Mean ± SD (L)	SD (L)	SRD (L)	SRD%	CV%
ATS/ERS standard and ATS/ERS mean	FEV_1_	246	3.27 (2.78, 3.84)	3.36 ± 0.80	0.14	0.40	10.34	3.73	251	3.24 (2.74, 3.87)	3.34 ± 0.78	0.10	0.29	8.59	3.10
	FVC		3.98 (3.38, 4.68)	4.06 ± 0.93	0.11	0.31	7.74	2.80		3.86 (3.33, 4.57)	4.03 ± 0.93	0.09	0.26	6.42	2.32
Mean of all, Mean of 3 largest, and Peak effort	FEV_1_	440	3.26 (2.76, 3.82)	3.34 ± 0.78	0.13	0.37	9.85	3.56	463	3.20 (2.74, 3.86)	3.30 ± 0.75	0.10	0.29	8.69	3.14
	FVC		3.94 (3.32, 4.55)	4.03 ± 0.90	0.11	0.30	7.47	2.70		3.83 (3.31, 4.55)	3.99 ± 0.90	0.10	0.29	7.15	2.58

ATS/ERS refers to the current selection method recommended by the American Thoracic and European Respiratory Societies. Data for descriptive statistics are presented as mean ± 1 standard deviation (SD) or median (interquartile range, IQR). While the repeatability criteria are different for the mean of all, mean of three largest and peak effort selection methods, the primary analysis dataset is for subjects in both visits and all selection methods. Therefore, the maneuvers included across these selection methods for intra-session comparisons for this dataset are the same. An illustration of how the descriptive statistics vary for datasets where subjects are not in both visits and not in all selection methods are presented in the Supplemental Materials secondary analyses. SD = measurement error taken as root mean square error (standard deviation) from fitted linear mixed effects models; SRD = repeatability or smallest real difference = SDx2.77; SRD% = SRDx100/mean across both visits for outcome measure; CV% = (SD/mean) × 100 across both visits for outcome measure.

### Inter-session variability

Inter-session descriptive statistics and variability are provided in Table 5 across selection methods. Like the results for intra-session variability, all estimates of measurement error, precision and repeatability for FEV_1_ and FVC were similar across selection methods. The difference in mean values for FEV_1_ and FVC from Visit 1 to Visit 2 were all less than 30 mL; CV% ranged from 2.16 to 2.95% for FEV_1_ and 1.61 to 2.11% for FVC (Table 5). Between Visit 1 and Visit 2, while the range of CV% values for FEV_1_ and FVC across selection criteria are similar, it can be noted that the overall magnitude of CV% values decreased in Visit 2.

**Table 5:** Inter-session descriptive statistics and variability by selection method for forced vital capacity (FVC) and forced expiratory volume in one second (FEV_1_) (n = 156; 78 per visit).

Selection method	Variable	Visit 1		Visit 2		SD (L)	SRD (L)	SRD%	CV%	ICC (95% CI)
		Median (IQR) (L)	Mean ± SD (L)	Median (IQR) (L)	Mean ± SD (L)					
ATS/ERS standard	FEV_1_	3.32 (2.84, 3.87)	3.41 ± 0.76	3.30 (2.80, 3.89)	3.40 ± 0.76	0.08	0.23	6.68	2.41	0.98 (0.96, 0.99)
	FVC	4.03 (3.44, 4.74)	4.10 ± 0.91	3.92 (3.38, 4.57)	4.09 ± 0.93	0.07	0.18	4.47	1.61	0.99 (0.98, 0.99)
ATS/ERS mean	FEV_1_	3.25 (2.76, 3.75)	3.33 ± 0.76	3.21 (2.73, 3.85)	3.32 ± 0.76	0.08	0.23	6.99	2.52	0.98 (0.96, 0.98)
	FVC	3.96 (3.39, 4.64)	4.02 ± 0.90	3.84 (3.31, 4.53)	4.01 ± 0.92	0.08	0.21	5.33	1.92	0.99 (0.98, 0.99)
Mean of all	FEV_1_	3.27 (2.74, 3.78)	3.33 ± 0.76	3.21 (2.74, 3.85)	3.32 ± 0.75	0.07	0.20	5.99	2.16	0.98 (0.97, 0.99)
	FVC	3.93 (3.36, 4.64)	4.03 ± 0.90	3.86 (3.32, 4.52)	4.01 ± 0.90	0.06	0.18	4.47	1.62	0.99 (0.98, 0.99)
Mean of 3 largest	FEV_1_	3.31 (2.81, 3.84)	3.39 ± 0.77	3.29 (2.79, 3.94)	3.38 ± 0.76	0.08	0.22	6.47	2.34	0.98 (0.97, 0.99)
	FVC	4.02 (3.41, 4.69)	4.09 ± 0.91	3.96 (3.41, 4.60)	4.08 ± 0.91	0.07	0.18	4.46	1.61	0.99 (0.98, 0.99)
Peak effort	FEV_1_	3.31 (2.82, 3.81)	3.38 ± 0.78	3.25 (2.79, 3.83)	3.35 ± 0.74	0.10	0.27	8.17	2.95	0.97 (0.95, 0.98)
	FVC	4.00 (3.37, 4.57)	4.05 ± 0.92	3.93 (3.40, 4.54)	4.06 ± 0.92	0.09	0.24	5.86	2.11	0.98 (0.97, 0.99)

ATS/ERS refers to the current selection method recommended by the American Thoracic and European Respiratory Societies. Data for descriptive statistics are presented as mean ± 1 standard deviation (SD) and median (interquartile range, IQR). SD = measurement error taken as root mean square error (standard deviation) from fitted linear mixed effects model; SRD = repeatability or smallest real difference (SRD) = SDx2.77; SRD% = SRDx100/mean across both visits for outcome measure; CV% = (SD/mean) × 100 across both visits for outcome measure; ICC = intraclass correlation coefficient; 95% CI = 95% confidence interval (lower, upper).

Overall, selection method and visit number were found to be statistically significantly associated with FVC (selection criteria: F = 27.08, *p* < 0.001; visit number: F = 4.84, *p* = 0.03) and FEV_1_ (selection criteria: F = 23.52, *p* < 0.001; visit number: F = 4.64, *p* = 0.03). The average differences in outcome measures (FEV_1_ or FVC) between each selection method relative to the model reference level (ATS/ERS standard) are provided in Table 6. Compared to the ATS/ERS standard selection method for reporting spirometric indices, the alternative selection methods all resulted in an average decrease in values reported for FEV_1_ and FVC ranging from about 10–80 mL less than the standard. Interaction plots for visit and criteria were explored for FEV_1_ and FVC; however, the results did not demonstrate evidence for interaction, so an interaction term was not included in the fitted models (see Supplemental Figure S1 for interaction plots).

**Table 6:** Effect estimates from fitted linear mixed effects models for each forced vital capacity (FVC) and forced expiratory volume in one second (FEV_1_).

Selection method (fixed effect in fitted mixed model)	Response variable	Average difference in response variable relative to model reference level (ATS/ERS standard) (L)	*p* value
ATS/ERS mean	FEV_1_	− 0.08	< 0.001
	FVC	− 0.08	< 0.001
Mean of all	FEV_1_	− 0.08	< 0.001
	FVC	− 0.07	< 0.001
Mean of 3 largest	FEV_1_	− 0.02	0.06
	FVC	− 0.01	0.25
Peak effort	FEV_1_	− 0.04	< 0.001
	FVC	− 0.04	< 0.001

The effect estimate here represents the average difference in outcome variable (FEV_1_ or FVC) between the alternative selection method versus the American Thoracic and European Respiratory Societies (ATS/ERS) standard method. A *p* value < 0.05 indicates the selection method was statistically significantly different from the ATS/ERS selection method.

## Discussion

The present study sought to evaluate four alternative selection methods for the reporting of FEV_1_ and FVC, as compared to the current ATS/ERS standard, with the goal of identifying the method that produced the least intra- and inter-session variability. The target question behind this work was whether or not alternative methods for selecting and reporting spirometric indices could provide more representative results of the test session as evidenced by thorough consideration of intra-session variability beyond the ATS/ERS standard repeatability threshold. Motivating this work was the recent ATS/ERS technical statement^4^ as well as a re-consideration of measurement error associated with spirometry^7^.

Contrary to our hypothesis, we observed similar measurement error, precision and repeatability for FEV_1_ and FVC across the five different selection methods in our sample of healthy non-smoking young adults for both intra- and inter-session analyses. We interpret these findings to suggest that under rigorously controlled laboratory conditions, and following recommended methods to select acceptable maneuvers, various methods for selecting valid data perform equally well with respect to minimizing variability and maximizing repeatability. Furthermore, relative to the ATS/ERS standard method, our four alternative selection methods resulted in lower FVC and FEV_1_ values (Table 6), but only by 0.01–0.08 L; this was less than the measurement errors across all fitted models (intra- and inter-session) regardless of which selection method was used.

### Intra-session variability

From a design perspective, it is important to note that all participants attempted at least eight consecutive maneuvers irrespective of achievement of any repeatability criterion with fewer maneuvers. This is an important distinction from prior work in this area that terminated testing within 3 or 5 maneuvers when repeatability criteria were met^9,13–17,20,21,23,41–46^. This approach facilitated consideration of alternative selection methods that included all acceptable maneuvers (Table 1) as well as allowed us to understand when, over a session, minimum and maximum values occurred for FEV_1_ and FVC (Fig. 1). Whether selecting only three or all available valid trials, the CV% for FEV_1_ and FVC ranged from 3.6 to 3.7% and 2.7 to 2.8% on Visit 1, respectively, with slight improvement on Visit 2 (Table 4). Although there appears some difference between methods as well as visit, these are approximately within the established accuracy tolerance of ± 3.0% (± 2.5% device, ± 0.5% calibration syringe;^4^).

Heat map analysis of the min/max occurrence of FEV_1_ and FVC over all trials for each patient made several trends apparent (Fig. 1). First, while minimum values appear to have occurred more commonly near the beginning of a session, maximum values were more likely to be interspersed across subjects’ attempted maneuvers for both FEV_1_ and FVC. This is particularly the case for the second session, whereas one might argue that maximum values show some clustering towards the center of attempted maneuvers during the first visit. Taken together, this trend suggests that learning effects may not play a role beyond initial trials. Second, both minimum and maximum FEV_1_ and FVC values commonly occurred during maneuvers that were not accepted, excluding these from further consideration in analyses. These data would seem to support earlier recommendations of including a practice trial at the beginning of a test session^16,21^ which is not currently recommended by the ATS/ERS^4^.

### Inter-session variability

The results of our mixed-effects model found that selection methods did not differ in a clinically meaningful way as a function of visit number despite participants reporting spirometry was easier to perform on their second visit. The lack of an observed difference across selection methods appears to reflect the stability of the measurement as indicated by the absence of any systemic bias due to visit or the presence of outliers (see Bland Altman plots, Supplemental Materials Figure S2). Further, ICCs for FEV_1_ and FVC using any selection method were ≥ 0.97 indicating excellent test–retest reliability. ICCs alone, however, do not fully capture the responsiveness of a particular method and other metrics such as the SRD are recommended^47^.

To illustrate an example, consider a controlled human inhalation-exposure study where the change in FEV_1_ from pre- to post-exposure to an agent is used to indicate a health effect. If the pre-exposure FEV_1_ is 3.41 L (using the mean of the sample for example), the post-exposure FEV_1_ would need to decrease beyond the observed SRD for the specific selection method, which for the ATS/ERS method was 230 mL or 6.7% (Table 5), for the reduction in FEV_1_ to be considered significant. If using an alternative method such as ‘mean of all’, FEV_1_ would need to decrease by 200 mL or 6.0%. As evidenced by Table 5 (and Table S6 in the Online Supplement that utilized the full sample), SRD values are minimally different across methods. Similar results across selection methods and their stability across visits may also be attributable to research design elements that minimized potential sources of error that could be controlled (e.g., environment, time of day, instructions, etc.). In addition, the participants appeared to have adhered to all instructions between visits and reported similar sleep quality and mood state prior to both visits.

### Strengths

Unlike cardiovascular screening tests (e.g., blood pressure), spirometry may be more prone to error given its dependence on individual factors such as patient effort and technician instruction. Great efforts have been made to standardize spirometry performance^4^ and technician training^48^ to minimize these sources of error. Still, there continues to be calls to action to enhance spirometry performance and data quality^49,50^. The present study was designed with these concerns in mind and implemented a variety of actions to ensure data were acquired and analyzed with great rigor. Unlike many prior studies in this area^13,14,16,21,41,46^, we used a single trained technician to acquire all data and provide all verbal standardized instruction and feedback. We took great care in screening our participants to ensure their lifestyle and behavioral characteristics were considered. Each maneuver was individually inspected by a NIOSH course instructor with over 10 years’ experience performing and evaluating spirometry as reflected by our thorough description of test and trial characteristics (Table 3). From a data analysis perspective, we designed a comprehensive strategy to evaluate measurement error and precision and utilized a mixed model approach to support our interpretation. The latter has not been previously performed in related literature evaluating multiple selection methods for spirometric variables which have relied upon practical or qualitative interpretations^6,15,17^. Conservatively, we delimited our primary analyses to the 60% of our sample that had valid data across methods and visits though sensitivity analyses on the larger sample confirmed our findings. (Secondary analyses provided in the Supplemental Materials).

### Limitations and future directions

Despite these strengths, our total sample size was relatively small in comparison to most of the related literature in this area^9,13,15,17,20,41,46^, with some exceptions^6,14,16,45^, reflective of our intended single site and technician approach. As such, we used a convenience sample like several other studies^13,17^, drawn primarily from our campus community. Our sample was comprised predominantly of women (71.0%) which appears dissimilar to early studies comprised predominantly of men^51,52^ or even sex distribution^17,46^. However, many earlier studies failed to report sex^9,14,43,45^ making this comparison difficult. Similarly, many earlier studies also did not report the race or ethnicity of their participants^6,14,16,20,41–43,46,51,52^ or were comprised exclusively of Caucasians^9,17,21,44^. Of those reporting race and ethnicity, only Wise et al.^15^ had a diverse sample similar to the present study albeit comprised of older current smokers with airflow obstruction. The latter point underscores that our results may only be applicable to our population of interest which are healthy young adults. Further validation is required to determine if similar results would be obtained in a healthy independent cohort. It should be noted that both intra- and inter-session variability may likely be affected by disease and individual responsiveness to exposure. Though the present study was focused on minimizing variability, we recognize that individual variability may afford unique clinical insight. For example, Veit et al.^53^ found that patients with interstitial lung disease who had greater daily FVC variability were at increased risk for disease progression. Still, separating variability attributable to measurement error versus disease is of utmost importance.

Despite substantial effort to ensure quality maneuvers (see Table 3), we recognize there is inherent bias in this approach. Other investigators have explored novel unbiased quality assurance indicators, such as real-time monitoring of exhaled breath volatile organic compounds during respiratory maneuvers^54,55^. These and other efforts to minimize bias are important future areas of research. Lastly, we decided a priori to focus on specific selection methods for reporting FEV_1_ and FVC, all but “peak effort” included a fixed volume rather than percentage for establishing repeatability (Table 1). This repeatability criterion has also been the source of debate and investigation with some advocating for a percentage criterion^46^ as opposed to the current practice of fixed volume criterion^4^. Taken together, the many combinations (e.g., number of trials included, descriptive statistic, repeatability criterion, etc.) that could potentially be analyzed are considerable and beyond the scope of the present study and statistical power though represent important areas for future research.

### Summary and conclusion

Spirometry follows a workflow for selecting variables to report from a given test session proceeding from: (1) determining individual trial acceptability, (2) to repeatability of trials within session, and finally (3) to reported variables representing that session’s trials. The current standard is to follow recommendations from ATS/ERS for this workflow. The goal of this work was to investigate whether alternative methods for selecting and reporting spirometric indices may provide more representative results of the test session as compared to the ATS/ERS standard. We hypothesized that selection methods that were not restricted to only including trials up to when repeatability was first established would confer less variability within a test session, and therefore less variability between test sessions. In our population of young healthy non-smoking adults in controlled laboratory conditions, the present study did not identify an optimal selection method (i.e., least variability, greatest repeatability) for acquiring and reporting FEV_1_ and FVC variables. However, there may be some potential benefit in including a practice trial prior to initiating a test session to avoid technical errors. Taken together, these results suggest that once repeatability criteria are achieved, the selection of which approach to use for reporting spirometric variables may be of low clinical significance in a healthy population. Whether similar findings would be obtained in independent cohorts requires further validation. Irrespective of these findings, we recommend future studies be explicit in their description of spirometry acquisition and analysis to facilitate comparability with the published literature.

## Supplementary Information

Supplementary Information.
