# Supplementary material for: A comparison of alternative selection methods for reporting spirometric parameters in healthy adults
Source: Sci Rep. 2021 Jul 22;11:14945. doi: 10.1038/s41598-021-94120-9 (PMC8298454; doi:10.1038/s41598-021-94120-9)

Supplemental Material

Title: A comparison of alternative selection methods for reporting spirometric parameters in healthy adults

Authors:
Jennifer H. Therkorn[1]
Daniella R. Toto[2]
*Michael J. Falvo[1,3]

Affiliations:
[1]Airborne Hazards and Burn Pits Center of Excellence, War Related Illness and Injury Study Center, VA New Jersey Health Care System; East Orange, NJ
[2]School of Osteopathic Medicine, Rowan University; Stratford, NJ
[3]Departments of Pharmacology, Physiology & Neuroscience and Physical Medicine & Rehabilitation, Rutgers New Jersey Medical School; Newark, NJ

***Correspondence:**
Michael J. Falvo, PhD
VA NJ Health Care System
385 Tremont Ave
East Orange, NJ 07018
800-248-8005
Michael.Falvo@va.gov

Sample R code for the ATS/ERS selection method

The ensuing code is provided for the ATS/ERS selection method as it was the most complex to code. Code for other selection criteria followed a modified version:

```
# To begin with the selection of maneuvers for intra-session comparison, first any patient visits with less than two trials are filtered out
criteria1.step1 <- accepted.data %>%
  group_by(patientID,visit) %>%
  filter(n()>2)

# Variables are created to identify repeatability criteria: the cumulative max for FVC and FEV1, cumulative second max for FVC and FEV1, sum of FVC and FEV1 (to be used for selecting other parameters of interest)
criteria1.step2 <- criteria1.step1 %>%
    mutate(nxtFVC = map_dbl(.x = seq_along(FVC), ~ ifelse(.x==1,NA,FVC[order(-FVC[1:.x])[2]])),
        nxtFEV1 = map_dbl(.x = seq_along(FEV1), ~ ifelse(.x==1,NA,FEV1[order(-FEV1[1:.x])[2]])),
        maxFVC = cummax(FVC),
        maxFEV1 = cummax(FEV1),
        sumFVC_FEV1 = (FVC + FEV1)))

# We define two variables that we will use in the next step to help us identify if each maneuver meets repeatability (the difference between the cumulative max and cumulative second max for FEV1 and FVC); we also define a variable for the ratio of max FEV1/FVC
criteria1.step2$rep_FEV1 <- (criteria1.step2$maxFEV1- criteria1.step2$nxtFEV1)
criteria1.step2$rep_FVC <- (criteria1.step2$maxFVC- criteria1.step2$nxtFVC)
criteria1.step2$ratio <- (criteria1.step2$maxFEV1/ criteria1.step2$maxFVC)

# First, any subjects not able to achieve repeatability across their maneuvers are filtered out. Then, a repeatability indicator variable is created that sequenced along each row of maneuver data indicating “YES” if repeatability established (rep_FEV1 and rep_FVC less than or equal to 0.15), or “NO” if this condition not met. Then, for each subject and visit, all rows were selected starting at their first row up to and including the first repeatable maneuver after at least three maneuvers performed.
(criteria1.step3 <- criteria1.step2 %>%
    filter(any(rep_FEV1<=0.15 & rep_FVC<=0.15 & trial>2)) %>%
    mutate(repeatable = ifelse((rep_FEV1<=0.15 & rep_FVC<=0.15 & trial>2),"YES","NO")) %>%
    slice(1: min(which(repeatable == "YES"))))

# Finally, FEV1, FVC and all other parameters were selected for inter-session comparisons from the set of repeatable maneuvers selected above for intra-session comparisons
criteria1.FEV1 <- criteria1.step3 %>%
  slice_max(FEV1,n=1,with_ties=FALSE)

criteria1.FVC <- criteria1.step3 %>%
  slice_max(FVC,n=1,with_ties=FALSE)

criteria1.other <- criteria1.step3 %>%
  slice_max(sumFVC_FEV1,n=1,with_ties=FALSE)
```

Secondary analyses

Table S1. Intra-session descriptive statistics: FVC and FEV_1

Criteria	Variable	Visit 1			Visit 2		
		N	Median (IQR)	Mean ± SD	N	Median (IQR)	Mean ± SD
**Dataset: all subjects regardless of complete data across visits or selection methods**							
**ATS/ERS standard & ATS/ERS mean**	**FEV_1**	317	3.33 (2.82, 3.93)	3.39 (0.80)	354	3.11 (2.60, 3.80)	3.24 (0.79)
	**FVC**	317	4.02 (3.43, 4.78)	4.11 (0.93)	354	3.72 (3.15, 4.54)	3.92 (0.96)
**Mean of all, Mean of 3 largest**	**FEV_1**	544	3.29 (2.79, 3.89)	3.36 (0.77)	619	3.13 (2.62, 3.79)	3.22 (0.77)
	**FVC**	544	4.00 (3.37, 4.70)	4.07 (0.90)	619	3.72 (3.16, 4.52)	3.90 (0.93)
**Peak Effort**	**FEV_1**	543	3.31 (2.76, 3.91)	3.37 (0.80)	612	3.14 (2.63, 3.84)	3.23 (0.77)
	**FVC**	543	4.00 (3.33, 4.70)	4.07 (0.92)	612	3.74 (3.16, 4.53)	3.91 (0.92)
**Dataset: subjects in both visits but not in all selection methods (due to repeatability)**							
**ATS/ERS standard & ATS/ERS mean**	**FEV_1**	283	3.24 (2.75, 3.80)	3.32 (0.79)	289	3.23 (2.73, 3.85)	3.30 (0.78)
	**FVC**	283	3.90 (3.32, 4.67)	4.04 (0.94)	289	3.82 (3.30, 4.66)	4.02 (0.95)
**Mean of all, Mean of 3 largest**	**FEV_1**	499	3.24 (2.75, 3.80)	3.31 (0.77)	524	3.20 (2.74, 3.85)	3.28 (0.74)
	**FVC**	499	3.87 (3.30, 4.59)	4.02 (0.91)	524	3.80 (3.31, 4.64)	4.00 (0.91)
**Peak Effort**	**FEV_1**	481	3.28 (2.76, 3.86)	3.36 (0.80)	503	3.20 (2.73, 3.87)	3.30 (0.78)
	**FVC**	481	4.00 (3.32, 4.65)	4.05 (0.91)	503	3.85 (3.27, 4.59)	3.99 (0.90)

Table S2. Intra-session descriptive statistics: PEF and FEF_{25-75}

Criteria	Variable	Visit 1			Visit 2		
		N	Median (IQR)	Mean ± SD	N	Median (IQR)	Mean ± SD
**Dataset: all subjects regardless of complete data across visits or selection methods**							
**ATS/ERS standard & ATS/ERS mean**	**PEF**	317	6.88 (5.74, 8.03)	7.02 (2.06)	354	7.00 (5.84, 8.31)	7.24 (2.15)
	**FEF_{25-75}**	317	3.44 (2.72, 4.39)	3.60 (1.11)	354	3.35 (2.67, 4.19)	3.46 (1.06)
**Mean of all, Mean of 3 largest**	**PEF**	544	6.74 (5.69, 7.90)	6.97 (2.03)	619	6.96 (5.84, 8.24)	7.18 (2.08)
	**FEF_{25-75}**	544	3.43 (2.77, 4.31)	3.56 (1.06)	619	3.36 (2.66, 4.14)	3.44 (1.04)
**Peak Effort**	**PEF**	543	6.81 (5.77, 7.94)	7.05 (2.07)	612	7.02 (5.87, 8.25)	7.21 (2.10)
	**FEF_{25-75}**	543	3.48 (2.76, 4.37)	3.60 (1.11)	612	3.40 (2.65, 4.22)	3.46 (1.06)
**Dataset: subjects in both visits but not in all selection methods (due to repeatability)**							
**ATS/ERS standard & ATS/ERS mean**	**PEF**	283	6.68 (5.56, 7.97)	6.92 (2.12)	289	7.09 (6.00, 8.50)	7.38 (2.10)
	**FEF_{25-75}**	283	3.35 (2.71, 4.21)	3.50 (1.05)	289	3.38 (2.67, 4.22)	3.45 (1.01)
**Mean of all, Mean of 3 largest**	**PEF**	499	6.67 (5.52, 7.88)	6.91 (2.07)	524	7.08 (6.08, 8.29)	7.33 (2.03)
	**FEF_{25-75}**	499	3.36 (2.76, 4.22)	3.50 (1.01)	524	3.39 (2.69, 4.18)	3.45 (0.99)
**Peak Effort**	**PEF**	481	6.73 (5.79, 7.92)	7.06 (2.11)	503	7.11 (6.23, 8.37)	7.45 (2.08)
	**FEF_{25-75}**	481	3.50 (, 2.76, 4.37)	3.59 (1.09)	503	3.46 (2.66, 4.25)	3.51 (1.08)
**Dataset: subjects in both visits and in all selection methods (primary data set)**							
**ATS/ERS standard & ATS/ERS mean**	**PEF**	246	6.74 (5.78, 7.98)	7.02 (2.10)	251	7.11 (6.23, 8.65)	7.52 (2.11)
	**FEF_{25-75}**	246	3.48 (2.73, 4.29)	3.58 (1.07)	251	3.46 (2.71, 4.28)	3.52 (1.02)
**Mean of all, Mean of 3 largest**	**PEF**	440	6.70 (5.77, 7.88)	7.01 (2.07)	463	7.10 (6.26, 8.35)	7.43 (2.05)
	**FEF_{25-75}**	440	3.49 (2.78, 4.32)	3.57 (1.04)	463	3.45 (2.68, 4.25)	3.50 (1.02)
**Peak Effort**	**PEF**	440	6.70 (5.77, 7.88)	7.01 (2.07)	463	7.10 (6.26, 8.35)	7.43 (2.05)
	**FEF_{25-75}**	440	3.49 (2.78, 4.32)	3.57 (1.04)	463	3.45 (2.68, 4.25)	3.50 (1.02)

Table S3. Intra-session variability by selection method

Criteria	Variable	Overall Mean	Visit 1					Visit 2				
			N	SD	SRD	SRD%	CV%	N	SD	SRD	SRD%	CV%
**Dataset: all subjects regardless of complete data across visits or selection methods**												
**ATS/ERS standard and mean**	**FEV_1**	3.31	317 maneuvers, 99 subjects	0.12	0.33	10.00	3.61	354 maneuvers, 110 subjects	0.10	0.28	8.60	3.11
	**FVC**	4.01	317 maneuvers, 99 subjects	0.11	0.30	7.47	2.70	354 maneuvers, 110 subjects	0.09	0.25	6.12	2.21
**Mean of all, mean of 3 largest**	**FEV_1**	3.28	544 maneuvers, 99 subjects	0.11	0.32	9.68	3.50	619 maneuvers, 110 subjects	0.11	0.29	8.95	3.23
	**FVC**	3.98	544 maneuvers, 99 subjects	0.11	0.29	7.35	2.65	619 maneuvers, 110 subjects	0.10	0.27	6.84	2.47
**Peak Effort**	**FEV_1**	3.30	543 maneuvers, 100 subjects	0.12	0.32	9.79	3.53	612 maneuvers, 109 subjects	0.11	0.30	8.97	3.24
	**FVC**	3.98	543 maneuvers, 100 subjects	0.11	0.29	7.40	2.67	612 maneuvers, 109 subjects	0.10	0.28	6.95	2.51
**Dataset: subjects in both visits but not in all selection methods (due to repeatability)**												
**ATS/ERS standard and mean**	**FEV_1**	3.31	283 maneuvers, 90 subjects	0.12	0.33	10.08	3.64	289 maneuvers, 90 subjects	0.10	0.28	8.48	3.06
	**FVC**	4.03	283 maneuvers, 90 subjects	0.11	0.30	7.42	2.68	289 maneuvers, 90 subjects	0.09	0.26	6.34	2.29
**Mean of all, mean of 3 largest**	**FEV_1**	3.30	499 maneuvers, 90 subjects	0.12	0.32	9.70	3.50	524 maneuvers, 90 subjects	0.10	0.28	8.55	3.09
	**FVC**	4.01	499 maneuvers, 90 subjects	0.11	0.29	7.30	2.63	524 maneuvers, 90 subjects	0.10	0.28	7.03	2.54
**Peak Effort**	**FEV_1**	3.33	481 maneuvers, 87 subjects	0.12	0.33	9.80	3.54	503 maneuvers, 87 subjects	0.11	0.29	8.75	3.16
	**FVC**	4.02	481 maneuvers, 87 subjects	0.11	0.30	7.48	2.70	503 maneuvers, 87 subjects	0.10	0.29	7.16	2.59

SD = measurement error taken as root mean square error from fitted linear mixed effects model

Repeatability or smallest real difference (SRD) = SDx2.77

SRD% = SRDx100/mean across both visits for outcome measure

CV% = (SD/ mean)x100 across both visits for outcome measure

Table S4. Inter-session descriptive statistics

Criteria	Variable	Visit 1			Visit 2		
		N	Median (IQR)	Mean ± SD	N	Median (IQR)	Mean ± SD
**Dataset: subjects in both visits but not in all selection methods (due to repeatability)**							
**ATS/ERS standard**	**FEV_1**	90	3.28 (2.82, 3.87)	3.38 (0.75)	90	3.29 (2.79, 3.87)	3.36 (0.76)
	**FVC**	90	3.99 (3.40, 4.76)	4.09 (0.92)	90	3.86 (3.37, 4.57)	4.07 (0.94)
**ATS/ERS mean**	**FEV_1**	90	3.22 (2.74, 3.75)	3.30 (0.75)	90	3.18 (2.71, 3.81)	3.28 (0.75)
	**FVC**	90	3.89 (3.33, 4.66)	4.01 (0.91)	90	3.77 (3.30, 4.54)	3.99 (0.93)
**Mean of all**	**FEV_1**	90	3.22 (2.73, 3.78)	3.30 (0.75)	90	3.18 (2.73, 3.82)	3.28 (0.75)
	**FVC**	90	3.88 (3.33, 4.64)	4.02 (0.91)	90	3.81 (3.32, 4.54)	3.99 (0.92)
**Mean of 3 largest**	**FEV_1**	90	3.28 (2.80, 3.83)	3.36 (0.76)	90	3.25 (2.76, 3.91)	3.34 (0.76)
	**FVC**	90	3.89 (3.36, 4.71)	4.07 (0.93)	90	3.90 (3.38, 4.64)	4.06 (0.93)
**Peak effort**	**FEV_1**	87	3.33 (2.78, 3.83)	3.38 (0.80)	87	3.30 (2.78, 3.90)	3.36 (0.76)
	**FVC**	87	4.01 (3.35, 4.61)	4.07 (0.93)	87	4.00 (3.35, 4.65)	4.07 (0.92)

Table S5. Inter-session descriptive statistics: PEF and FEF_{25-75}

Criteria	Variable	Visit 1			Visit 2		
		N	Median (IQR)	Mean ± SD	N	Median (IQR)	Mean ± SD
**Dataset: subjects in both visits but not in all selection methods (due to repeatability)**							
**ATS/ERS standard**	**PEF**	90	6.84 (5.70, 8.17)	7.09 (2.02)	90	7.40 (6.18, 8.47)	7.53 (2.10)
	**FEF_{25-75}**	90	3.41 (2.77, 4.32)	3.57 (0.99)	90	3.43 (2.86, 4.31)	3.56 (1.00)
**ATS/ERS mean**	**PEF**	90	6.56 (5.69, 7.95)	6.91 (2.04)	90	7.07 (5.90, 8.31)	7.33 (2.06)
	**FEF_{25-75}**	90	3.35 (2.73, 4.22)	3.48 (1.00)	90	3.31 (2.69, 4.24)	3.44 (0.97)
**Mean of all**	**PEF**	90	6.66 (5.80, 7.80)	6.92 (2.02)	90	7.01 (6.10, 8.18)	7.31 (2.06)
	**FEF_{25-75}**	90	3.35 (2.74, 4.24)	3.46 (1.00)	90	3.35 (2.66, 4.20)	3.43 (0.97)
**Mean of 3 largest**	**PEF**	90	6.65 (5.69, 7.97)	7.02 (2.02)	90	7.21 (6.12, 8.46)	7.42 (2.09)
	**FEF_{25-75}**	90	3.42 (2.76, 4.27)	3.53 (0.98)	90	3.40 (2.78, 4.24)	3.51 (0.98)
**Peak effort**	**PEF**	87	7.63 (6.50, 8.56)	7.73 (2.11)	87	7.80 (6.70, 8.97)	8.10 (2.14)
	**FEF_{25-75}**	87	3.65 (2.71, 4.46)	3.60 (1.08)	87	3.45 (2.64, 4.22)	3.54 (1.03)
**Dataset: subjects in both visits and in all selection methods (primary data set)**							
**ATS/ERS standard**	**PEF**	78	6.86 (5.95, 7.98)	7.17 (2.01)	78	7.46 (6.30, 8.61)	7.66 (2.12)
	**FEF_{25-75}**	78	3.52 (2.77, 4.39)	3.65 (1.01)	78	3.53 (2.89, 4.37)	3.64 (1.00)
**ATS/ERS mean**	**PEF**	78	6.60 (5.84, 7.96)	7.00 (2.04)	78	7.21 (6.18, 8.57)	7.49 (2.07)
	**FEF_{25-75}**	78	3.50 (2.75, 4.27)	3.55 (1.02)	78	3.50 (2.73, 4.28)	3.52 (0.99)
**Mean of all**	**PEF**	78	6.79 (5.93, 7.93)	7.05 (2.01)	78	7.12 (6.31, 8.48)	7.47 (2.06)
	**FEF_{25-75}**	78	3.54 (2.76, 4.26)	3.54 (1.02)	78	3.49 (2.67, 4.26)	3.51 (0.98)
**Mean of 3 largest**	**PEF**	78	6.75 (6.00, 8.02)	7.14 (2.01)	78	7.34 (6.34, 8.64)	7.60 (2.10)
	**FEF_{25-75}**	78	3.60 (2.77, 4.36)	3.61 (1.00)	78	3.56 (2.82, 4.29)	3.59 (0.99)
**Peak effort**	**PEF**	78	7.35 (6.44, 8.56)	7.68 (2.09)	78	7.82 (6.67, 8.93)	8.05 (2.15)
	**FEF_{25-75}**	78	3.63 (2.76, 4.47)	3.60 (1.02)	78	3.44 (2.63, 4.22)	3.52 (0.97)

Table S6. Inter-session variability by selection method

Criteria	Variable	N	Mixed effects ANOVA F value for visit	Mixed effects ANOVA p value for visit	Mean	SD	SRD	SRD%	CV%	ICC (95% CI)
**Dataset: subjects in both visits but not in all selection methods (due to repeatability)**										
**ATS/ERS standard**	**FEV_1**	180 (90 each visit)	1.00	0.32	3.37	0.08	0.22	6.52	2.36	0.98 (0.97, 0.99)
	**FVC**	180 (90 each visit)	2.23	0.14	4.08	0.06	0.18	4.38	1.58	0.99 (0.99, 0.99)
**ATS/ERS mean**	**FEV_1**	180 (90 each visit)	0.96	0.33	3.29	0.08	0.22	6.80	2.45	0.98 (0.97, 0.99)
	**FVC**	180 (90 each visit)	1.95	0.17	4.00	0.07	0.21	5.13	1.85	0.99 (0.98, 0.99)
**Mean of all**	**FEV_1**	180 (90 each visit)	1.16	0.28	3.29	0.07	0.19	5.91	2.14	0.98 (0.97, 0.99)
	**FVC**	180 (90 each visit)	3.12	0.08	4.01	0.06	0.17	4.36	1.57	0.99 (0.99, 0.99)
**Mean of 3 largest**	**FEV_1**	180 (90 each visit)	0.87	0.35	3.35	0.08	0.22	6.44	2.33	0.98 (0.97, 0.99)
	**FVC**	180 (90 each visit)	1.42	0.24	4.07	0.07	0.18	4.43	1.60	0.99 (0.99, 0.99)
**Peak effort**	**FEV_1**	174 (87 each visit)	1.08	0.30	3.37	0.10	0.27	8.14	2.94	0.97 (0.95, 0.98)
	**FVC**	174 (87 each visit)	0.03	0.86	4.07	0.09	0.24	5.89	2.13	0.98 (0.97, 0.99)

SD = measurement error taken as root mean square error from fitted linear mixed effects model
Repeatability or smallest real difference (SRD) = SDx2.77
SRD% = SRDx100/mean across both visits for outcome measure
CV% = (SD/ mean)x100 across both visits for outcome measure
ICC = intraclass correlation coefficient
95% CI = 95% confidence interval (lower, upper)

Table S7. Bland-Altman bias and limits of agreement for forced vital capacity (FVC) and forced expiratory volume in one second (FEV_1) by selection method across two visits. These values are plotted in the Bland Altman plots of the supplemental materials below (Figure S2).

Criteria	Variable	Bias (95% CI)	Lower Limit (95%CI)	Upper Limit (95% CI)
**ATS/ERS Standard**	**FEV_1**	0.01 (-0.02, 0.05)	-0.31 (-0.37, -0.25)	0.33 (0.27, 0.40)
	**FVC**	0.02 (-0.01, 0.05)	-0.24 (-0.29, -0.19)	0.28 (0.23, 0.33)
**ATS/ERS Mean**	**FEV_1**	0.01 (-0.03, 0.05)	-0.32 (-0.38, -0.25)	0.34 (0.27, 0.41)
	**FVC**	0.02 (-0.02, 0.05)	-0.28 (-0.35, -0.22)	0.32 (0.26, 0.38)
**Mean of all**	**FEV_1**	0.01 (-0.02, 0.05)	-0.27 (-0.32, -0.21)	0.30 (0.24, 0.35)
	**FVC**	0.02 (-0.01, 0.05)	-0.23 (-0.29, -0.18)	0.28 (0.23, 0.33)
**Mean 3 largest**	**FEV_1**	0.01 (-0.02, 0.05)	-0.30 (-0.36, -0.24)	0.32 (0.26, 0.38)
	**FVC**	0.01 (-0.02, 0.04)	-0.25 (-0.30, -0.19)	0.27 (0.22, 0.32)
**Peak effort**	**FEV_1**	0.02 (-0.02, 0.07)	-0.36 (-0.44, -0.29)	0.41 (0.33, 0.49)
	**FVC**	0.00 (-0.04, 0.04)	-0.34 (-0.41, -0.27)	0.33 (0.27, 0.40)

Figure S1. FEV_1 and FVC interaction plots for visit number vs. selection method (primary dataset, n = 78).

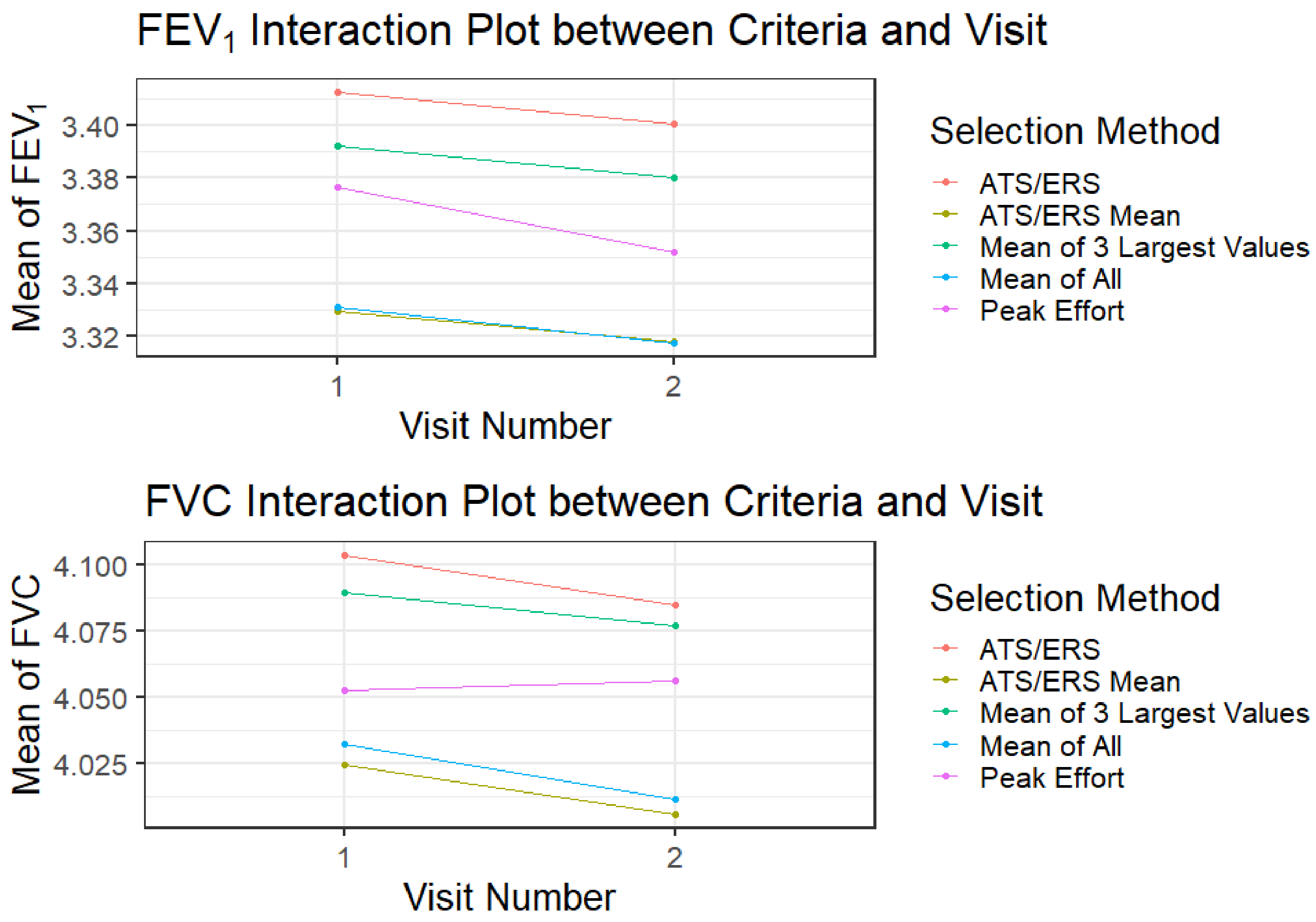

Figure S2. Bland Altman plots for FEV_1 and FVC by selection method (n = 78). Vertical and horizontal axes represent the difference (Visit 1 – Visit 2) and average values, respectively. The bias and 95% limits of agreement (dashed black horizontal lines) are plotted in red and blue dashed lines, respectively. The values used to construct these plots are reported in the Supplemental Material Table S7.

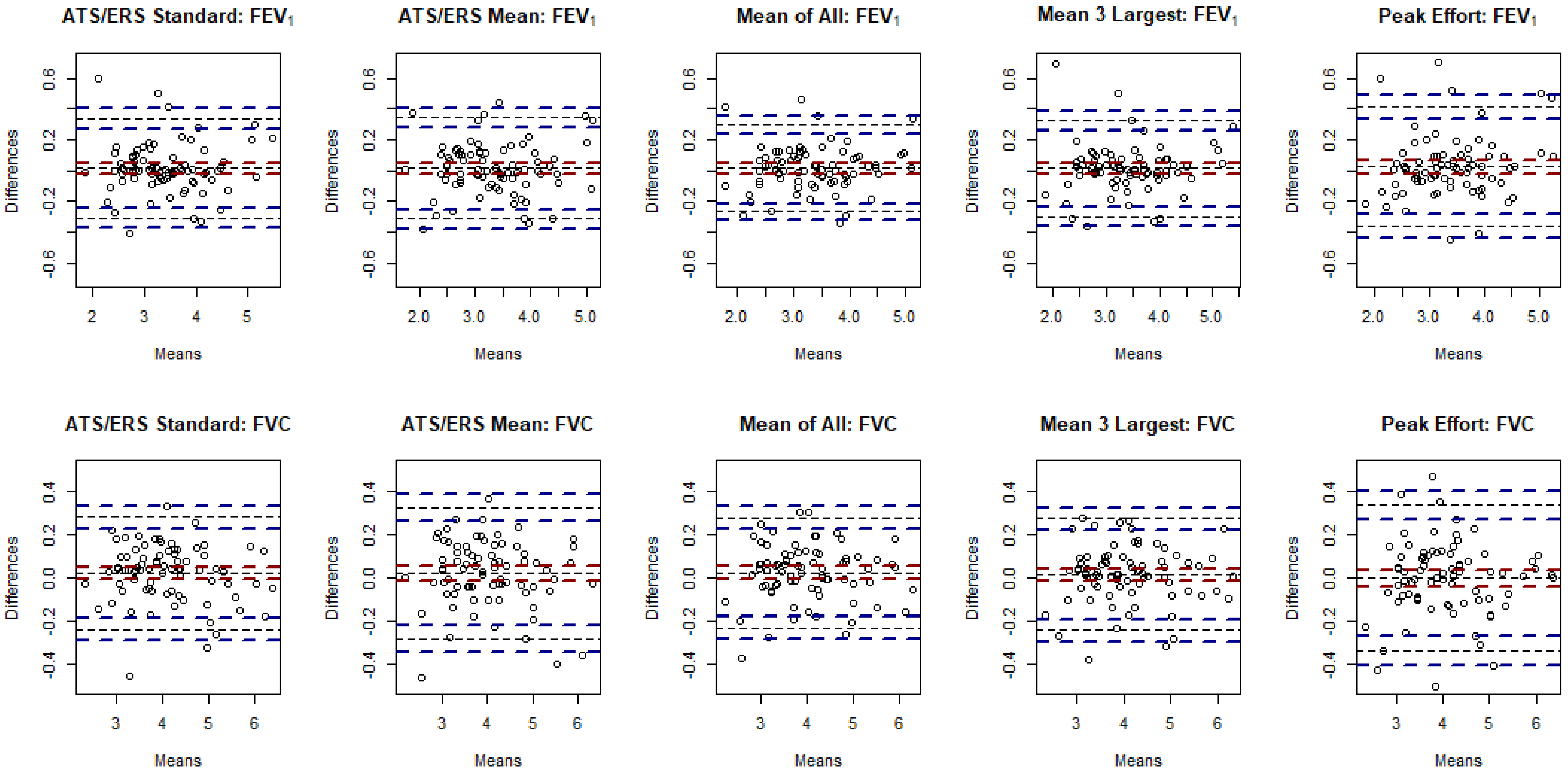

Supplement: Supplementary file 1 — Supplementary Information. [file 41598_2021_94120_MOESM1_ESM.pdf]
